# Integration of Online Treatment Into the “New Normal” in Mental Health Care in Post–COVID-19 Times: Exploratory Qualitative Study

**DOI:** 10.2196/21344

**Published:** 2020-10-08

**Authors:** Joyce J P A Bierbooms, Monique van Haaren, Wijnand A IJsselsteijn, Yvonne A W de Kort, Milou Feijt, Inge M B Bongers

**Affiliations:** 1 Tranzo Tilburg School of Social and Behavioral Sciences Tilburg University Tilburg Netherlands; 2 Mental Healthcare Eindhoven Eindhoven Netherlands; 3 Eindhoven University of Technology Eindhoven Netherlands

**Keywords:** online treatment, sustainability, mental health care, COVID-19

## Abstract

**Background:**

The COVID-19 pandemic has necessitated an immediate and large-scale uptake of online treatment for mental health care. However, there is uncertainty about what the “new normal” in mental health care will be like in post–COVID-19 times. To what extent will the experiences gained during the pandemic influence a sustainable adoption and implementation of online mental health care treatment in the future?

**Objective:**

In this paper, we aim to formulate expectations with regard to the sustainability of online mental health care after COVID-19.

**Methods:**

In an interview study, 11 mental health care professionals were asked about their experiences and expectations for the future. Participants were recruited from a mental health care organization in the Netherlands. The interviews took place between April 7-30, 2020, at the peak of the COVID-19 crisis in the Netherlands. The data were analyzed using a thematic coding method.

**Results:**

From the interviews, we learn that the new normal in mental health care will most likely consist of more blended treatments. Due to skill enhancement and (unexpected) positive experiences with online treatment, an increase in adoption is likely to take place. However, not all experiences promise a successful and sustainable upscaling of online treatment in the future. Mental health care professionals are learning that not all clients are able to benefit from this type of treatment.

**Conclusions:**

Sustainable upscaling of online mental health care requires customized solutions, investments in technology, and flexibility of mental health care providers. Online treatment could work for those who are open to it, but many factors influence whether it will work in specific situations. There is work to be done before online treatment is inherently part of mental health care.

## Introduction

In recent months, the COVID-19 pandemic and subsequent governmental regulations in the Netherlands have urged mental health care providers to accommodate an immediate and large-scale uptake of online treatment. Up to that point, many mental health care professionals were still quite hesitant, despite the proven benefits, to use online treatment options [1,2]. Previous studies have shown that the adoption of online treatment is influenced by a variety of factors, such as a lack of digital skills among both practitioners and clients, technical issues, the assumption that technology-mediated treatment may not allow for real interpersonal contact, and (lack of) prior experiences regarding the potential added value of online treatment [3-5].

Many questions currently are being raised about what the “new normal” of society will constitute, if and when COVID-19 has run its course. This also applies to mental health care. Both optimism and concerns have been expressed about the acceptance of online treatment as a new normal in mental health care [6-10]. There is optimism about improving the accessibility of mental health care by upscaling online treatment and accommodating clients to receive therapy in a way that easily fits into their daily lives. This may empower clients, foster their self-efficacy, and enable them to engage in their treatment (more) independently of time and place (eg, [8,10]). However, concerns are expressed that not everyone will benefit equally from this game changer. Specifically, vulnerable mental health clients may lack the digital skills, cognitive ability, motivation, and/or resources to partake successfully in online digital treatment, and are at risk of being disconnected from the care they need (eg, [7,9]).

Online treatment as part of the new normal in mental health care will—and must—depend on mental health care professionals’ and clients’ experiences regarding their use of online treatment during the COVID-19 pandemic [10]. These experiences are therefore important sources of feedback that will help anticipate the effects of this period of “forced upscaling” on the sustainability of online treatment in mental health care and the new normal that we will find ourselves in.

Based on mental health care professionals’ experiences during the COVID-19 pandemic, the current paper seeks to discuss the expectations regarding the sustainability of online treatment in mental health care when it is no longer necessitated by COVID-19 regulations.

## Methods

### Design

We used an exploratory qualitative study design in which we interviewed mental health care professionals about their experiences with online treatment during the COVID-19 period and their expectations regarding the new ways of working in mental health care when this crisis is controlled.

### Context

In the Netherlands, mental health care is provided within a system of frontline services by general practitioners; primary and secondary mental health care is provided by specialized mental health care providers. Funding depends on the type of treatment that is required and can be provided by the national government (long-term secondary mental health care), health insurance companies (primary and short-term secondary mental health care), or the local government (youth mental health care). Online treatment is seen as an important development in mental health care and is stimulated accordingly. For example, while waiting lists can be long in mental health care, health insurance companies have made arrangements with mental health care professionals to provide online treatment to people on the waiting list. This is fully funded although people are not yet registered as clients [11].

This study was conducted at Stichting Geestelijke Gezondheidszorg Eindhoven en de Kempen (GGzE), a mental health care provider in Eindhoven, the Netherlands. GGzE has approximately 2200 employees (including management and supporting staff) and provides clinical and ambulatory mental health care to more than 20,000 clients. Mental health care is provided either in group therapy or individually and can be face to face or online. In 2019, GGzE started an online treatment team providing full online treatment trajectories (ie, using videoconferencing, online modules, and messaging services). This online treatment team currently has a caseload of approximately 200 clients with light to complex psychological and psychiatric problems.

### Participants

For the interviews, we recruited 9 mental health care professionals and 2 eHealth support staff employees from GGzE. In the sample of mental health care professionals, 5 had recently started working online, and 4 were already experienced, having used online treatment tools for more than 1 year. The latter group comprised those who specifically chose to be an online therapist and were therefore already positive about it before they started, in contrast to the therapists who were recently “forced” to start with online treatment since face-to-face therapy was not possible. During the COVID-19 pandemic, online treatment services consisted of videoconferencing, online treatment modules, and messaging services using a secured communication platform. Whereas a broad range of other online treatment tools are available (eg, virtual reality, self-monitoring apps), the upscaling of online treatment in the context of the COVID-19 crisis primarily concerns the aforementioned tools. During the COVID-19 period, an average of 1060 therapy sessions were conducted online per week versus 59 online consultations per week in the first 6 weeks of 2020.

The participants were approached after obtaining consent from their manager. They were provided with an invitational email in which the purpose of the interview was explained; then volunteers were contacted by the researcher. The purpose of the interview was explained once again, and the participants were asked to sign an informed consent form.

### Data Collection

Data collection took place between April 7-30, 2020, when the COVID-19 crisis was at its peak and mental health care professionals had been working online between 3-6 weeks. Mental health care professionals who were new to using online treatment were asked about their experiences related to performing treatment online, their skill improvement, and their intentions to use online treatment in the future. Mental health care professionals who had been using online treatments for >1 year were asked about the barriers they had encountered prior to becoming an online therapist, their current experiences, and their strategies for establishing rapport with their clients. The supporting staff was asked which questions and concerns they encountered while supporting professionals with online treatment tool use. All participants were asked about their expectations regarding the continued use of online psychological treatment in post–COVID-19 times.

### Data Analysis

The interviews were recorded and transcribed verbatim. The data were analyzed using a thematic coding approach [12]. Themes were identified based on the purpose of the study. Subsequently, all fragments of the data were coded and assigned to subthemes.

## Results

### Overview

**Figure 1:**
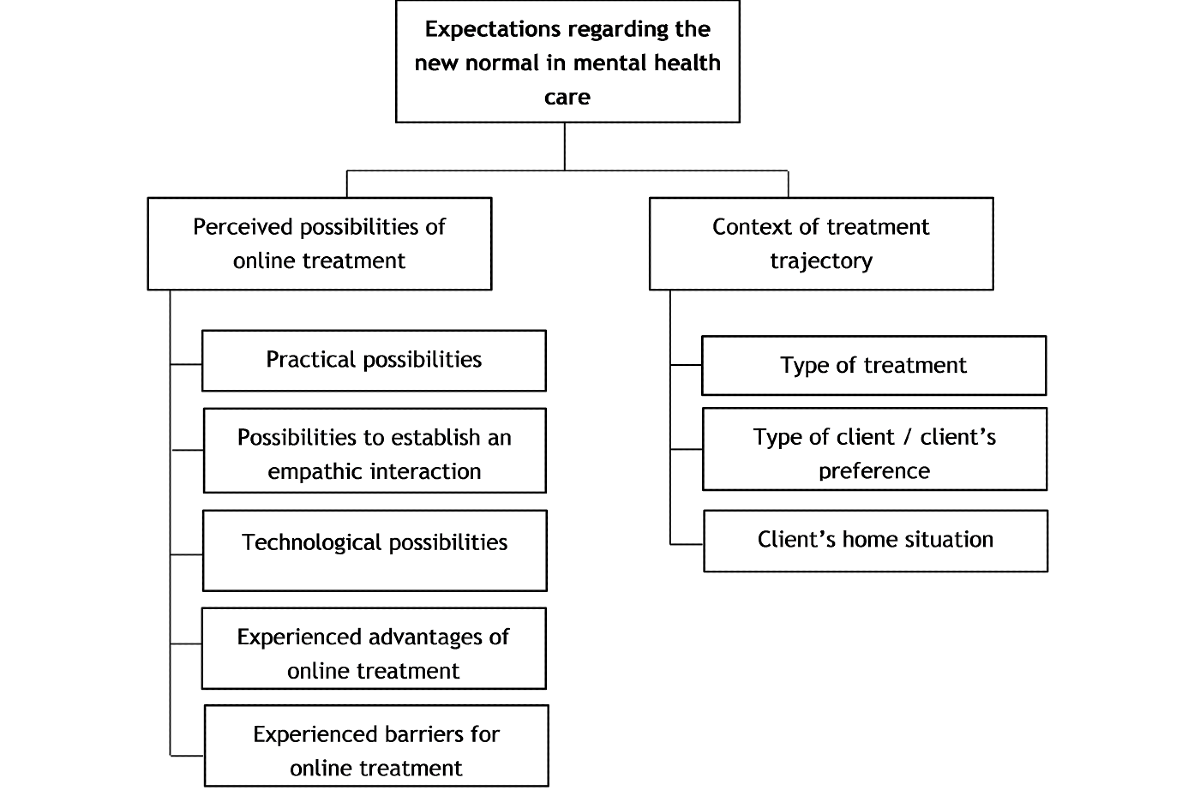
Themes and subthemes.

From the data analysis, two main themes could be derived regarding the role of online treatment in the new normal in mental health care: the perceived possibilities of online treatment and the context of a treatment trajectory. The data revealed several subthemes that represent the issues that contribute to this new normal based on current experiences during the COVID-19 period from the perspective of the mental health care professionals themselves and how they perceive their clients to experience online treatment. Figure 1 illustrates the main themes and subthemes.

The results of the interviews provide preliminary insight into what the new normal in mental health care and the role of online treatment could look like. There were a number of (unexpected) positive experiences, but there were also concerns. On the one hand, the results suggest that because important barriers such as a lack of skills and experience have now been lowered, online treatment will be used more frequently. On the other hand, some professionals also expect that they, or their colleagues, will revert to regular face-to-face treatment as soon as possible, since a number of professionals and clients perceive regular face-to-face treatment as the only way to create a therapeutic alliance. In general, we found that the participants in this study who were already experienced online practitioners were more confident about the continuation of online treatment in the future compared to others.

### Perceived Possibilities of Online Treatment

Overall, the results show that mental health care professionals come to understand that much more is possible in online treatment than what they had initially expected, for example, drawing something on paper or sharing one’s screen to go through an online module together with a client. Mental health care professionals also report that their clients tend to be more open when they are in their home environment and that genuine empathic contact is indeed possible in remote communication. These positive effects surprised the mental health care professionals who started delivering online treatment during the COVID-19 period; the experienced online practitioners were already convinced of these possibilities. One respondent predicted that because clients are now experiencing the benefits of online treatment, there is a significant chance that they will continue to ask for it, which will to a large extent determine whether online treatment options will be integrated into the new normal. Furthermore, mental health care professionals expect that they will more frequently consider online treatment as an alternative now that they are aware of its potential value. Reported examples of such added value include the reduction in travel time, an increase in self-efficacy and activation in clients, more openness by clients when they are in their home environment, and increased accessibility to treatment due to flexibilities in time and place. This, in turn, leads to more frequent, shorter consultations, which adds to a higher level of connectedness between client and therapist.

I do this by writing a message each morning asking my client how he slept and then I can give some suggestions to get through the day. [...] I noticed, and this is surprising to me, that this delivers epistemic trust from my client towards me.

P1

In a face-to-face meeting, you tend to fill the 45 minutes you planned, despite the fact that you only need 20 minutes, because your client came all the way over to your office. When I have an online meeting with my client, I can much more easily finish the meeting after 20 minutes when we have nothing else to talk about anymore.

P4

The future position of online treatment in mental health care will also be determined by the technological conditions required to conduct online treatments. Mental health care professionals report having experienced high levels of frustration over technical issues they encountered while trying to get their online environments to work. These problems are felt by both new and experienced online therapists. In addition, clients do not always possess the necessary technical devices or a robust internet connection, nor do they always have an appropriate and quiet place with sufficient privacy where they can talk to their therapist. There is also the issue of confidence in the security of the system, which in some cases withholds professionals, but mainly clients, from wanting to perform therapy online. Moreover, some clients receive therapy without their family/relatives’ knowledge, which makes it difficult to engage in online sessions from home.

Especially now that the kids are at home it is very difficult for clients to find a quiet place to talk to me. Sometimes they are calling by telephone from the car because they could not find a proper place at home.

P6

A struggle that is also mentioned, mostly by the more unexperienced online therapists, is that it is very difficult to connect at an interpersonal level during an online interaction, such as videoconferencing. Nonverbal cues can be easily missed, which requires a number of additional questions and explicit feedback moments from a therapist. Moreover, participants stated multiple times that it can be very difficult to react adequately to clients’ emotions in an online therapeutic setting.

It can be very difficult to react to a client’s emotions, for example, when someone starts to cry, I find it difficult to react when I am not in the same room. There is literally a distance.

P9

### Context of a Treatment Trajectory

According to mental health care professionals, the continued use of online treatment in the future will depend on the type of treatment and the type of clients—there will not be a one-size-fits-all solution. On this issue, both experienced and unexperienced online mental health care practitioners agree. The resilience that is now shown by a number of clients in responding to online treatment does not apply to every one of them. According to the participants, not all clients are equally susceptible to online treatments. The general experience is that group therapy does not work well online, nor do therapy sessions with multiple participants from the social network of a client. In addition, Eye Movement Desensitization and Reprocessing (EMDR) is considered close to impossible to perform online by some professionals. Interestingly, others report unexpected positive experiences regarding complex therapies such as EMDR and imagery rescripting. For those with positive experiences with these complex therapies, the benefits that were mentioned earlier (eg, reduction of travel time) will probably determine whether these forms of therapy will be continued remotely after the COVID-19 period. There is also no consensus regarding the necessity to do at least the intakes in a face-to-face setting. In particular, therapists who are more experienced in online treatment tend to claim they have very effective online intakes for various complex mental health care problems. This may imply that the number of online intakes might increase to at least some extent in comparison to the pre–COVID-19 situation, due to experiences gained in this period.

Some colleagues argue that an intake cannot be done online, but I have very positive experiences with online intakes.

P7

## Discussion

### Overview

As the COVID-19 mitigation measures are slowly being lifted in the Netherlands, mental health care organizations have started to think about expanding possibilities beyond regular face-to-face treatment. Numerous pleas, opinions, and discussions regarding the continuation of online treatment in mental health care are being prompted in professional literature, on professional network platforms, and on social media. The question we raised at the beginning of this paper becomes increasingly urgent with each step mental health care takes in liberalizing COVID-19 measures: what new normal will we adopt regarding online treatment in mental health care?

### Expectations

Based on therapists’ experiences with online treatment during the COVID-19 period, we can create a clearer image of the possibilities of online treatment in mental health care and how a sustainable increase in the use of online tools can be reached. As it is clear that experience and skill enhancement will lead to fewer barriers among mental health care professionals to use online treatment tools [13-15], there will likely be more online treatment in the near future than there was before COVID-19. We also argue that some of the positive experiences will convince even therapists who were previously resistant toward online treatment to consider an online session when this saves time and is more convenient for the client. A recent study shows that, in most cases, the perceived advantages regarding online treatment outweigh the disadvantages [3]. However, we also have reason to believe that in some cases online therapy may not work. There are issues that are more difficult to overcome, such as clients having an inadequate environment without proper (technological) facilities to receive online treatment or clients for whom it is difficult to open up to this concept. The conviction that a real therapeutic alliance can be achieved through remote communication does not seem to come simply by gaining experience in using online treatment. Additionally, frequent users of online treatment and therapists who are generally positive about the possibilities prefer to see people face to face in certain situations. The bottom line is that mental health care delivery in the new normal will most likely result in more blended forms of care. The sustainability of blended care requires giving space to diversity and being flexible in providing online treatment for those who benefit from it, while providing alternatives for those who do not.

### Limitations

This paper describes a small study with 11 participants from one particular mental health care organization. This means that further research is needed to strengthen the results. Despite the small sample size, data saturation was reached and many mental health care professionals also indicated that were disclosing ideas and experiences of their colleagues. Another limitation is that clients were not interviewed in this round. This was due to the limited amount of time in which we wanted to draw a preliminary overview of experiences with online treatment in mental health care. A focus on mental health care professionals was chosen because of the large impact the adoption among therapists has on a sustainable implementation of online treatment. The clients’ experiences that are described in this paper are reflections of practitioners based on their interactions with their clients about online treatment.

### Future Research

The sustainability of online treatment in mental health care will depend on the complex interplay of individual, social, organizational, and economic factors [16]. The exact implications of the COVID-19 crisis for online mental health care need further exploration taking into account the complexity of the situation. In our future work, we will investigate the sustainability of online treatment in mental health care in post–COVID-19 time in relation to the significant changes the pandemic and its aftermath will continue to impose upon mental health care. We will continue to work with mental health care organizations in addressing the challenges they face in effectively delivering online and blended forms of treatment, including skill enhancement in online treatment of mental health care professionals and investigating the acceptance and use of online treatment tools by both therapists and clients. In addressing these challenges, our focus is on innovative solutions aimed to optimize the accessibility, effectiveness, efficiency, and treatment quality of online treatment tools, including their various technological and user requirements as well as the organizational conditions that encourage a sustainable uptake of online treatment.

## Abbreviations

EMDR: Eye Movement Desensitization and Reprocessing
GGzE: Stichting Geestelijke Gezondheidszorg Eindhoven en de Kempen
